# dsRID: *in silico* identification of dsRNA regions using long-read RNA-seq data

**DOI:** 10.1093/bioinformatics/btad649

**Published:** 2023-10-23

**Authors:** Ryo Yamamoto, Zhiheng Liu, Mudra Choudhury, Xinshu Xiao

**Affiliations:** Bioinformatics Interdepartmental Program, University of California, Los Angeles, CA 90095-1570, United States; Department of Integrative Biology and Physiology, University of California, Los Angeles, CA 90095-7246, United States; Department of Integrative Biology and Physiology, University of California, Los Angeles, CA 90095-7246, United States; Bioinformatics Interdepartmental Program, University of California, Los Angeles, CA 90095-1570, United States; Department of Integrative Biology and Physiology, University of California, Los Angeles, CA 90095-7246, United States; Molecular Biology Institute, University of California, Los Angeles, CA 90095-1570, United States

## Abstract

**Motivation:**

Double-stranded RNAs (dsRNAs) are potent triggers of innate immune responses upon recognition by cytosolic dsRNA sensor proteins. Identification of endogenous dsRNAs helps to better understand the dsRNAome and its relevance to innate immunity related to human diseases.

**Results:**

Here, we report dsRID (double-stranded RNA identifier), a machine-learning-based method to predict dsRNA regions *in silico*, leveraging the power of long-read RNA-sequencing (RNA-seq) and molecular traits of dsRNAs. Using models trained with PacBio long-read RNA-seq data derived from Alzheimer’s disease (AD) brain, we show that our approach is highly accurate in predicting dsRNA regions in multiple datasets. Applied to an AD cohort sequenced by the ENCODE consortium, we characterize the global dsRNA profile with potentially distinct expression patterns between AD and controls. Together, we show that dsRID provides an effective approach to capture global dsRNA profiles using long-read RNA-seq data.

**Availability and implementation:**

Software implementation of dsRID, and genomic coordinates of regions predicted by dsRID in all samples are available at the GitHub repository: https://github.com/gxiaolab/dsRID.

## 1 Introduction

Cytosolic double-stranded RNAs (dsRNAs), upon recognition by dsRNA sensor proteins, can trigger innate immune responses (Cheng *et al.* 2007). This mechanism constitutes a primary means in human cells to defend against viral infections. However, dsRNAs are also generated endogenously, many of which may be candidate binding targets of cytosolic sensor proteins, such as MDA5, RIG-I, or PKR. Unwanted activation of antiviral signaling by endogenous dsRNAs is prevented at least partly by the Adenosine-to-Inosine (A-to-I) RNA editing. A-to-I editing is performed by the adenosine deaminase acting on RNA (ADAR) enzymes that bind to dsRNAs (Liddicoat *et al.* 2015, Nakahama and Kawahara 2023). Accumulating evidence suggests that A-to-I editing by ADAR and its binding to endogenous dsRNA affect dsRNA immunogenicity, implicated in cancer, autoimmune and inflammatory diseases (Wang *et al.* 2020, Li *et al.* 2022, Chan *et al.* 2023).

Identification of endogenous dsRNAs related to immunogenicity remains a major challenge. Since ADAR is a dsRNA-binding protein, A-to-I editing sites have been used as indicators of the existence of dsRNA regions. To this end, methods have been developed to leverage editing-enriched regions (EERs) to define endogenous dsRNA structures (Whipple *et al.* 2015, Blango and Bass 2016). This type of method may use all known editing sites, such as those cataloged in RNA editing databases (Kiran and Baranov 2010, Ramaswami and Li 2014, Mansi *et al.* 2021), to enable a comprehensive identification of possible dsRNAs. However, the resulting dsRNAs may not be specific to the samples under study. Alternatively, RNA editing sites identified in the samples at hand may be used in the analysis, with the risk of limited coverage as it is likely that only a subset of true editing sites are identified. Despite these limitations, EER-based methods are widely used computational approaches in identifying dsRNAs with potential relevance to innate immunity.dsRNA structures that undergo no or low-level RNA editing in a specific sample may escape from identification by EER-based methods (Reich and Bass 2019). Low RNA editing levels may result from regulation of ADAR activities or competition between RNA-binding proteins and ADAR (Rybak-Wolf *et al.* 2014, Quinones-Valdez *et al.* 2019). Such unedited dsRNAs may be potent activators of antiviral signaling. Thus, it is important to design methods for editing-independent identification of dsRNAs. Popular experimental methods to identify endogenous dsRNAs include J2 antibody pull down assays, such as dsRIP-seq and J2 fClLIP-seq (Kim *et al.* 2018, Gao *et al.* 2021). Other experimental methods (such as SHAPE or PARS) are available for global RNA structure analysis independent of RNA editing (Kertesz *et al.* 2010, Loughrey *et al.* 2014). In addition, protein–RNA binding profiling for dsRNA-binding proteins provides a basis to infer dsRNA regions (Rybak-Wolf *et al.* 2014, Bahn *et al.* 2015, Quinones-Valdez *et al.* 2019). However, most of the above experimental methods possess limited sensitivity due to technical challenges. Methods to detect dsRNA computationally in a high-throughput manner are highly desirable.

In this work, we developed a new approach, named double-stranded RNA Identifier (dsRID), to detect dsRNA regions in an editing-agnostic manner. This method is built upon a previous observation made by us and others that dsRNA structures may induce region-skipping in RNA-sequencing (RNA-seq) reads, an artifact likely reflecting intramolecular template switching in reverse transcription (Cocquet *et al.* 2006, Houseley and Tollervey 2010, Tardaguila *et al.* 2018, Liu *et al.* 2023). Leveraging this observation and long-read RNA-seq data, we constructed a machine-learning model that extracts features from mapped reads and outputs predictions of dsRNA regions. Using features related to region-skipping, dsRID achieved *in-silico* identification of dsRNA regions independent of editing with high accuracy. We applied this method to a few long-read RNA-seq data derived from Alzheimer’s disease (AD) and control samples, which predicted novel dsRNAs with low RNA editing levels.

## 2 Materials and methods

### 2.1 Datasets

AD long-read RNA-seq data was downloaded from PacBio (https://www.pacb.com/connect/datasets/). Long-read RNA-seq data of GM12878 cells and nine samples of human mid-frontal cortex (AD or controls) were downloaded from the ENCODE project (https://www.encodeproject.org/ accession numbers: ENCSR962BVU, ENCSR462COR, ENCSR169YNI, ENCSR257YUB, ENCSR690QHM, ENCSR316ZTD, ENCSR697ASE, ENCSR094NFM, ENCSR463IDK, and ENCSR205QMF). Reads in the fastq files were aligned using minimap2 according to the ENCODE standard parameters with the additional—cs flag for downstream analysis (Li 2021).

### 2.2 Dataset curation for training and validation

For each long-read RNA-seq dataset, we first defined positive regions and negative regions for model training. Positive regions were defined as those with known dsRNAs annotated by EER-based methods (see below) (Whipple *et al.* 2015, Blango and Bass 2016, Liu *et al.* 2023). Negative regions were randomly sampled regions non-overlapping with the positive regions and with a window size of 2500 nt. To prevent each region from having null feature values, both positive and negative regions were required to have at least six reads in total and at least one read with region-skipping. We matched the number of negative regions to the number of positive regions to balance the dataset.

### 2.3 Identification of EERs

Based on the approach suggested by Whipple *et al.* (Blango and Bass 2016, Reich and Bass 2019), we identified EERs using editing sites from REDIportal (Mansi *et al.* 2021). Regions were defined as editing enriched if there existed at least three editing sites in a 50-bp window. Subsequently, EERs that were within 1 kb from each other were merged. These regions were then structurally verified using RNAfold (Lorenz *et al.* 2011), and only dsRNAs with at least 200 bp stem length with up to 20% of mismatches, as well as an adjusted MFE ≤ −0.35, were retained. The adjusted MFE was calculated as the ratio between RNAfold-calculated MFE and the length of the folded sequence (multiplied by 100) (Zhang *et al.* 2006). The above cutoffs were chosen to enrich for long dsRNAs that are potentially strong substrates of MDA5 (Blango and Bass 2016, Ahmad *et al.* 2018).

### 2.4 Feature extraction

For each region of interest, we extracted features based on reads mapped to the region. The features were defined as follows:

Skip_ratio (i.e. skipping ratio): The number of reads that contained internal skipping divided by the total number of reads mapped to the region.

Len_skip (i.e. skipping length): Average length of skipped regions among reads with internal skipping. In the minimap2-generated bam file, the start and end of continuous cs tags indicating “∼” (internal skipping signal) were considered as the start and end sites of the skipped region. In alignments where the cs tag was not available, we used “N”s in the CIGAR strings as an indication of a skipped region. Following the determination of the start and end sites, we calculated their genomic distance in each read, and used the average value of this distance metric among all reads of a skipped region as the skipping length feature.

Group_num (i.e. number of skipping groups): The number of distinct skipping groups. The start and end sites of skips were grouped together when the sites were within 100 bp of each other. We assigned all sites to a group so that the left most site and right most site in each group were <100 bp away from the median of the same group. If the numbers of groups for the start sites and end sites were different, we took their average as the overall number of skipping groups from both ends.

Std_start, std_end: Standard deviation of the genomic coordinates corresponding to the start and end sites of skipped regions, respectively. For each skipping group identified above, we calculated the standard deviation of the start and end positions, respectively, across all reads. Standard deviations are then averaged across different skipping groups of each region.

Gc_skip: Average GC content of the skipped region across all mapped reads of the region.

Bp_start_*, bp_end_*: Occurrence frequency of two bases prior to and after each end of the skipped region, aiming to differentiate stochastic skipping from splicing that has specific splicing donor and acceptor sequences. All 16 di-nucleotides were tested for the start and end sites, respectively. Only the top five are shown in Fig. 2E.

### 2.5 Hyperparameter tuning using TPOT

We used TPOT (Le *et al.* 2020) for feature selection, model selection, and hyperparameter tuning in the dsRID model trained on the PacBio AD data. TPOT is an automated machine-learning optimization tool, which selects models for different sets of hyperparameters. TPOT-tuned parameters for the random forest classifier are shown below (Table 1). The scikit-learn software package was used to train the random forest classifier and other hyperparameters were set to their default values in the RandomForestClassifier function.

**Table 1.** Hyperparameters used to train random forest classifier tuned by TPOT. The first column indicates the name of the hyperparameter and the second column represents the hyperparameter value used in the model.

Hyperparameter name	Hyperparameters used
Split criteria	Gini index
Whether to use bootstrapped samples	Yes
Maximum depth of the tree	No depth limit
Minimum number of samples for splitting	2
Minimum number of samples for leaf nodes	4
Maximum fraction of features to be considered for splitting	20%
Number of trees	100

### 2.6 Permutation-based feature contribution analysis

To investigate the relative contribution of each feature to the overall model, we employed permutation-based feature contribution analysis using the random forest model trained with the PacBio AD dataset. First, we computed the variance explained by the model (*R*^2^) using a held-out validation dataset. Next, for each feature, we permuted its feature vector and recomputed *R*^2^ in the validation set. The decrease in the recomputed *R*^2^ relative to the original *R*^2^ was defined as the contribution score of the feature. We used the python package scikit-learn to conduct this procedure (Pedregosa *et al.* 2011).

### 2.7 Calculation of minimum free energy for each candidate region

For each candidate dsRNA region, we used RNAfold (Lorenz *et al.* 2011) in the Vienna RNA package to compute minimum free energy and its corresponding structure. RNAfold was run using default parameters and the -AMFE flag to compute adjusted minimum free energy.

### 2.8 Discovery of novel dsRNA regions

To discover novel dsRNA regions in each dataset, we analyzed windows spanning 2500 nt with a sliding step of 1250 nt across the genome. Those with at least six reads in total and at least one read with internal skipping were retained for feature extraction.

We ran dsRID using the random forest classifier trained on the PacBio AD dataset to compute the probability of forming a dsRNA in each region. For regions with more than 50% probability of being dsRNA and without an overlap with EER-based dsRNAs, we further examined their folded structures using RNAfold in order to classify them into candidate novel long dsRNAs or structured RNAs. Similarly, as in the identification of dsRNAs based on EERs (see above), novel long dsRNAs were required to have a stem length of ≥200 bp with ≤20% mismatches and an adjusted MFE ≤ −0.35. Candidates that did not meet the above requirements were called structured RNAs.

### 2.9 Calculation of A-to-I editing index

To analyze A-to-I RNA editing levels for each region we identified, we used editing sites published in the REDIportal database (Mansi *et al.* 2021) and RNAEditingIndexer to convert aligned reads to base-by-base pileups (Roth *et al.* 2019). We only included editing sites that were covered by more than three reads and calculated editing index of each region as the total number of *G* nucleotides divided by the sum of the numbers of *A* and *G* nucleotides.

## 3 Results

### 3.1 Overview of the dsRID method

**Figure 1.**
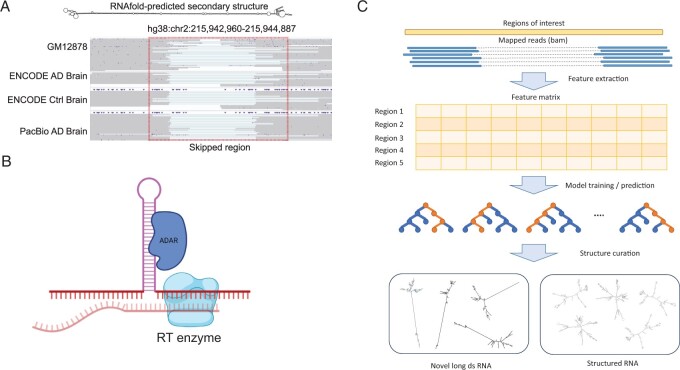
Overview of the dsRID method. (A) An example region showing internal skipping that occurs in multiple datasets. Top: RNAfold-predicted structure of the genomic region. Bottom: IGV plots of mapped reads from four datasets. Ctrl: control. (B) Schematic diagram showing the hypothesis of template skipping due to double-stranded structure and ADAR binding (created by Biorender). (C) Schematic diagram for the steps in dsRID

In a previous study with long-read RNA-seq data, we observed that many reads contained internal skipped regions that mimic spliced-out introns (Liu *et al.* 2023). However, such region-skipping is unlikely a result of splicing as they were not flanked by typical splice site sequences and the starts and ends of the skipped region did not align consistently across multiple reads (Fig. 1A). We hypothesized that this observation reflects reverse transcriptase (RT)-generated deletion artifacts in cDNAs. As previously reported, such artifacts may be caused by intramolecular template switching, a process where RT skips the hairpin structure of the template RNA (Cocquet *et al.* 2006, Houseley and Tollervey 2010, Tardaguila *et al.* 2018) (Fig. 1B).

Inspired by the above observation, we built a machine-learning model, dsRID, to predict whether certain transcripts form dsRNA structures using only features related to internal region-skipping in the long reads. The dsRID method consists of four main steps: feature extraction, training, prediction, and structure curation (Fig. 1C). After a standard read mapping procedure using minimap2 (Li 2021), we focused on regions (2500 nt in length) with at least six mapped reads and at least one read with internal skipping (hereafter referred to as candidate regions). We extracted a number of features from such regions, e.g. skipping ratio (calculated as the ratio of reads that contained internal skipping among all reads overlapping a region), skipping length (calculated as the average length of internal skipping harbored in all reads of a region), and standard deviations of the start and end positions of the skipped region (Section 2). For training purposes, we used previously curated dsRNA regions as a positive set, which were defined based on EERs (Section 2) (Blango and Bass 2016), and randomly sampled regions outside of the curated dsRNA as a negative set. Note that the random controls (2500 nt in length) were also required to have ≥6 mapped reads and ≥1 read with internal skipping. Thus, such controls may encompass regions with pre-mRNA splicing events.

Following feature extraction for both positive and negative sets, we trained binary classifier models, such as random forests, logistic regression, and support vector machines, to predict dsRNA regions (Fig. 1C). We used TPOT (Le *et al.* 2020) to tune the hyperparameters and select the model with the best performance (Section 2). In the prediction step, we applied the model to all candidate regions (as defined above) across the genome, excluding positive regions with curated dsRNAs (Section 2). Given the binary classification problem, we defined predicted “candidate dsRNA regions” as those that passed the threshold of 0.5 in the predicted probability. Next, in the step of structure curation, we applied RNAfold to evaluate the structures of these candidate dsRNAs, and identified them as “novel long dsRNAs” or generally “structured RNAs” (Section 2). Novel long dsRNAs, with a ≥ 200 bp stem region, are potentially immunogenic as substrates of dsRNA sensors, such as MDA5 (Ahmad *et al.* 2018). Generally structured RNAs do not possess such long dsRNA structures.

### 3.2 dsRID predicts dsRNA regions with high performance across several datasets

**Figure 2.**
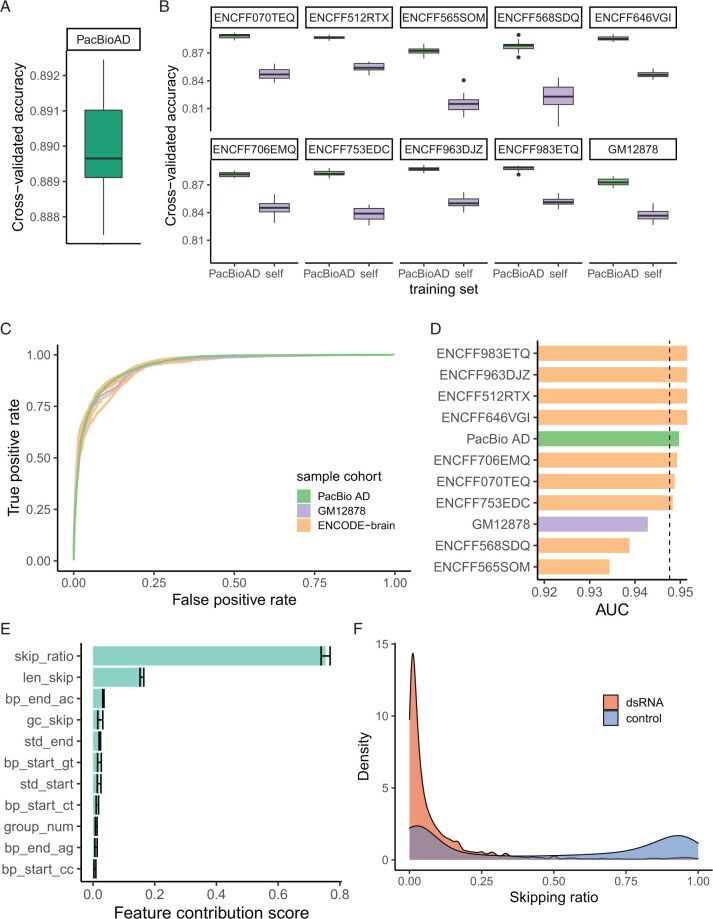
dsRID predicts dsRNA regions with high performance across several datasets. (A) Box plot showing 20-fold cross-validated accuracy of dsRID trained on PacBio-AD data. (B) Box plots showing cross-validated accuracy of dsRID for different datasets. *X*-axis indicates whether the model is trained on its own dataset (self) or the PacBio-AD data. (C) ROC showing the performance of dsRID trained on the PacBio-AD dataset. *Y*-axis represents true positive rate and *x*-axis represents false positive rate. (D) AUC of the ROC for each dataset. The datasets are color-coded as shown in (C). Dashed line indicates the mean of AUC scores across datasets. (E) Bar plot showing feature contribution score for each feature in the PacBio-AD-trained dsRID model (see Section 2). (F) Distribution of skipping ratios stratified by known dsRNA and controls

We first evaluated the performance of the model using long-read RNA-seq data derived from the brain sample of an AD patient generated by Pacific Biosciences (PacBio AD). We trained the model on 13 469 positive regions and the same number of randomly selected negative regions. We carried out 20-fold cross-validation and observed an accuracy of 89% (Fig. 2A). Next, we evaluated the performance of the method applied to other long-read RNA-seq datasets. Specifically, we used 10 ENCODE datasets generated from the GM12878 cells or frontal cortexes of healthy individuals or patients with AD. The number of regions used in the training step for each dataset is shown in Supplementary Fig. S1, which approximately correlated with the sequencing depth due to the coverage requirements in defining the candidate regions. It should be noted that the performance evaluation below included all predicted candidate dsRNAs (i.e. both novel long dsRNAs and structured RNAs).

We carried out 20-fold cross-validation for each dataset using two different models, the model trained using the same dataset and the one derived from the PacBio AD data that had the largest sequencing depth. When trained with each respective dataset, the average cross-validation accuracy was 84.1%. In contrast, this accuracy was 88.3% using the PacBio AD-trained model for each dataset (Fig. 2B). The enhanced accuracy in the latter case likely reflects the fact that the PacBio AD data had the highest sequencing depth and the most training regions to encompass a comprehensive dsRNA landscape (Supplementary Fig. S1).

To analyze how sequencing depth affects the performance of dsRID, we randomly subsampled the PacBio AD data (4.27 million reads originally) to mimic lower sequencing depth. We then trained the dsRID model using the subsampled datasets. As shown in Supplementary Fig. S2A, the number of dsRID-identified candidate dsRNA regions decreased with smaller number of reads. In addition, these numbers were generally lower than cases where the model trained on the full PacBio AD data was used on these subsampled datasets. To evaluate the accuracy of the models trained on subsampled data, we tested them using 5% of the original PacBio AD data, and repeated this process 20 times (Supplementary Fig. S2B). The accuracy of the models improved with larger subsampled sizes, and somewhat plateaued at around 1.5–2 million reads. Overall, the accuracy was relatively high, above 80% with very low sequencing depth (0.43 million). In addition, we also carried out similar procedures using the model trained on the original PacBio AD dataset but applied to the subsampled data (Supplementary Fig. S2C). In this case, the accuracy was consistently high (>88%). Based on the above results, we recommend that users collect at least 1.5–2 million reads for each dataset. The number of discoveries and accuracy will both increase with higher sequencing depth. For users without deeply sequenced samples for model training, we recommend using our pre-trained model of the PacBio AD data (provided with the dsRID package). For all analyses below, we used this pre-trained model since it is the best performing model overall.

We further evaluated the performance of our model on each dataset using receiver-operator curve (ROC) analysis and calculated the area under the curve (AUC). The average AUC across all datasets was 0.95 (Fig. 2C and D). In addition, we used precision–recall curves to evaluate the performance and calculated the area under the precision–recall curve (AUPRC). The average AUPRC across all cohorts was 0.94 (Supplementary Fig. S3). These results suggest that our model performs well in terms of both sensitivity and specificity.

To examine the relative importance of each feature, we performed a permutation-based feature contribution analysis (Section 2). We first computed the variance explained by the model (*R*^2^ value). This variance was then compared to that calculated by permuting each feature vector respectively. The reduction in *R*^2^ upon the permutation was defined as the contribution score of the corresponding feature. We observed that the skipping ratio had the highest contribution score (75.4%), followed by the length of skipping (15.9%) (Fig. 2E and F and Supplementary Fig. S4). Specifically, the positive dsRNA regions had a much lower skipping ratio than randomly sampled regions (Fig. 2F). This observation suggests that region-skipping due to dsRNA structures occurs randomly to a minor fraction of the cDNA molecules. In addition, the randomly sampled regions showed bimodally distributed skipping ratios, similar to the distribution of exon inclusion levels in splicing (Fig. 2F). Indeed, the random regions were significantly closer to (many overlapping with) known spliced junctions than positive regions in the training data (Supplementary Fig. S5, see Section 4).

### 3.3 Characterizations of novel dsRNA regions predicted by dsRID

**Figure 3.**
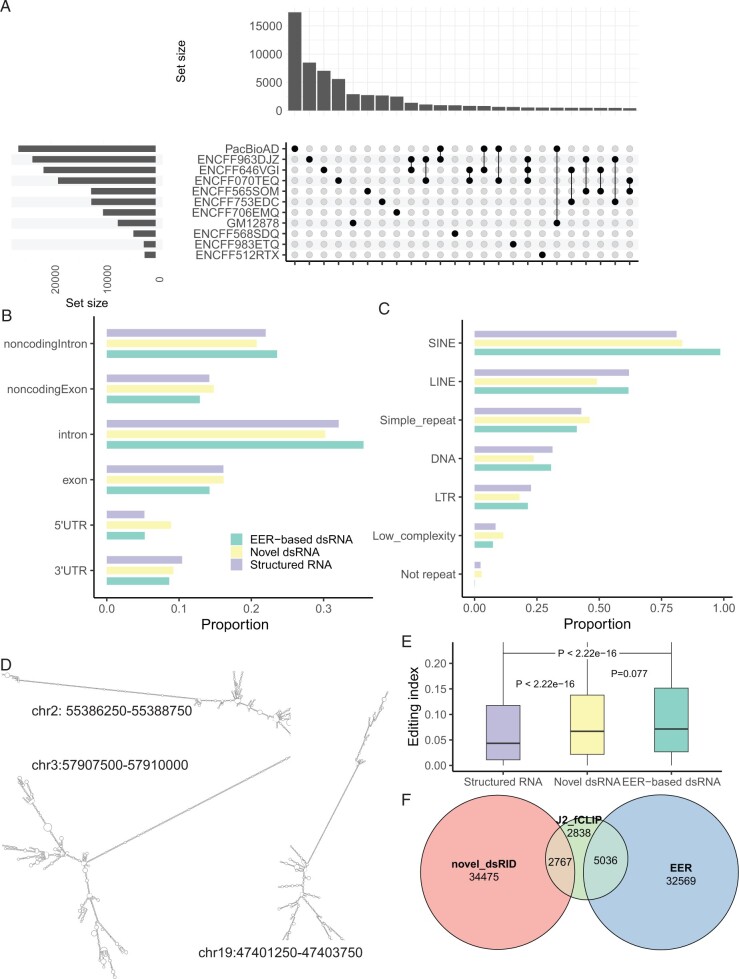
Characterization of novel dsRNA regions predicted by dsRID. (A) Upset plot showing the number of novel dsRNAs detected by dsRID in each dataset and the overlaps across different datasets. Bars on the left: the number of novel dsRNAs for each dataset, bars on the top: the number of novel dsRNAs that are unique to each dataset or shared between multiple datasets. (B) Proportion of EER-based dsRNA, novel long dsRNAs, or structured RNAs in different types of regions. Region categories are defined by Gencode v36 annotations. “noncodingintron” and “noncodingexon” groups represent intronic and exonic regions of non-coding RNAs. “intron,” “exon,” “3′UTR,” and “5′UTR” groups represent regions from coding genes. (C) Proportion of EER-based dsRNA, novel long dsRNAs, or structured RNAs in different types of repeats. (D) Example novel dsRNA structures and their genomic coordinates (hg38). (E) Editing index of EER-based dsRNAs, novel dsRNAs, or structured RNAs. *P*-values were calculated via Wilcoxon rank-sum tests. (F) Overlap between dsRID regions (including EER-based and novel dsRNAs) and J2 fCLIP-captured regions

A total of 82 266 candidate dsRNA regions (not present in the positive set for training) were identified across all 11 datasets (PacBio AD and 10 ENCODE datasets). As shown in Fig. 3A, the majority of candidate dsRNAs were unique to one dataset, which may reflect the fact that only a subset of true dsRNAs was captured in each dataset limited by sequencing depth. Alternatively (or additionally), this observation may be due to region-skipping occurring relatively randomly to structured regions. Among all candidate dsRNA regions, 32 391 were categorized as novel long dsRNA based on RNAfold, and 49 875 were denoted as structured RNAs (Section 2).

We next analyzed the characteristics of the union of all novel long dsRNAs from the 11 datasets. Similarly, as EER-based dsRNAs (used as positive training data by dsRID), novel dsRNAs most frequently overlapped with intronic regions compared to other regions. Interestingly, the novel dsRNAs were more enriched in 5′-UTRs relative to EER-based dsRNAs (Fig. 3B, Proportion test, *P* < 2.2e-16). This observation indicates that there may exist more dsRNA structures in 5′-UTRs than appreciated previously, which may have regulatory impacts, such as translational regulation (Leppek *et al.* 2018). Structured RNAs did not show substantial difference in their regional distributions relative to the EER-based or novel dsRNAs.

Furthermore, we analyzed the overlap of dsRNAs with different types of repetitive regions. As expected, most EER-based dsRNAs overlapped with SINE elements (Fig. 3C), reflecting the fact that they were derived from EERs enriched in Alu regions. Although dsRID does not impose bias on the types of regions from which to discover dsRNAs, the novel dsRNAs also had high enrichment in repetitive sequences, especially SINEs, consistent with the propensity of repetitive elements forming highly structured regions. Nonetheless, compared to EER-based dsRNAs, novel dsRNAs were significantly less enriched in SINEs (Proportion test, *P* < 2.2e-16), likely due to the editing-independent identification enabled by dsRID. Notably, structured RNAs also had enrichment in repetitive sequences, supporting that such RNAs have repeat-generated structures. The structures of a few example novel long dsRNAs are shown in Fig. 3D, which harbor extended double-stranded regions. Overall, the enrichment of novel dsRNAs in repetitive sequences supports the validity of their predicted existence.

Moreover, we investigated whether the novel dsRNAs were enriched with A-to-I RNA editing sites. We used human editing sites published in REDIportal and computed their editing ratios in the long-read RNA-seq data (Mansi *et al.* 2021). We observed a significant but modest positive correlation between the dsRID-predicted probabilities of dsRNAs and RNA editing index (Supplementary Fig. S6). This observation suggests that regions that are edited *in vivo* are more likely predicted as dsRNAs by our method. Nonetheless, compared to that of EER-based dsRNAs, the RNA editing index of novel dsRNAs is slightly lower (Fig. 3E). However, both novel and EER-based dsRNAs had significantly higher editing indexes than structured RNAs (Fig. 3E). Together, the above data support the validity of the predicted novel dsRNAs. Importantly, many novel dsRNAs discovered in this study may have low RNA editing levels, which may have been missed by previous methods built upon EERs.

Lastly, we examined whether dsRID predictions overlapped with dsRNAs captured by experimental methods. Specifically, we obtained dsRNAs identified by the J2 fCLIP-seq experiment in Hela cells (Kim *et al.* 2018). Despite the cell type differences, 73.3% of the J2-captured dsRNAs overlapped with dsRID dsRNAs (combining results from all datasets in this study, Fig. 3F). Among these dsRNA regions (7803 in total), 5036 were found in EERs and 2767 were novel long dsRNAs. In addition, more than 67 000 dsRNAs were included in dsRID (32 569 EERs and 34 475 novel), but not in the J2 fCLIP-seq, possibly reflecting limited sensitivity of the experiment or the fact that the dsRID results were combined from multiple human tissues and cell lines.

### 3.4 Comparative analysis of dsRNA in AD and controls detected by dsRID

**Figure 4.**
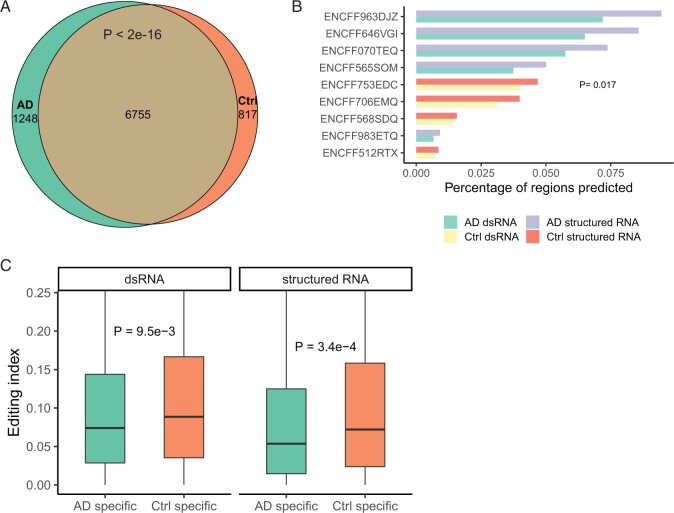
Comparative analysis of dsRNAs in AD and controls detected by dsRID. (A) Venn diagram showing the overlap between dsRNAs detected in AD and control samples. (B) Percentage of predicted dsRNAs or structured RNAs among all candidate regions analyzed for each dataset. (C) Editing index in AD-specific and control-specific dsRNAs or structured RNA regions. *P*-values were calculated by two-sided *t*-test to compare the editing index between AD and control-specific regions

To gain insights into the dsRNA profiles in AD, we conducted comparative analysis between AD and control brain samples from the ENCODE consortium. First, we asked whether the overall dsRNA (including both EER-based and novel dsRNAs) profiles were distinct between AD and controls. Among all candidate regions that were tested in both AD and control samples, 76.3% were identified as long dsRNAs in both groups, whereas 14.2% were specific to AD samples and 9.3% specific to controls. Proportion of AD-specific dsRNAs were significantly higher compared to control-specific dsRNAs (Fisher’s exact test, *P* < 2.2e-16, Fig. 4A). In addition, for each sample, we calculated the fraction of predicted novel dsRNAs and structured RNAs among all tested candidates. The AD samples showed a significantly higher novel dsRNA and structured RNA fractions (Wilcoxon rank-sum test, *P* = .017, Fig. 4B). Furthermore, the overall expression level of dsRNAs is higher in AD than in controls, suggesting higher production of dsRNAs in AD (Supplementary Fig. S7). In contrast, the AD-specific dsRNAs had lower editing index than control-specific dsRNAs (Fig. 4C). The above data suggest that although the overall editing level is lower in AD samples, the total production of dsRNAs is higher in AD (Fig. 4B and C and Supplementary Fig. S7).

## 4 Discussion

Obtaining dsRNA profiles *in silico* may greatly facilitate investigations of dsRNA-related innate immunity. In this study, we developed dsRID, a method to predict dsRNA regions using information captured in a single long-read RNA-seq dataset. The performance of dsRID is consistently high across several datasets, suggesting that the features included in dsRID reflect general characteristics of long-read RNA-seq data. dsRID identifies dsRNAs independent of RNA editing sites, in contrast to previous methods based on EERs (Whipple *et al.* 2015, Blango and Bass 2016). We applied dsRID to data generated from AD and control brain samples. Despite the limited sample size, dsRID enabled identification of many dsRNAs, with potentially distinct expression and editing profiles between AD and controls.

Given its editing-agnostic nature, dsRID has a unique advantage over editing-based approaches in enabling dsRNA discoveries for samples with low baseline editing. Certain disease conditions, such as psoriasis (Shallev *et al.* 2018), autism spectrum disorders (Tran *et al.* 2019), and schizophrenia (Choudhury *et al.* 2023) are known to have reduced RNA editing levels overall. In such scenarios, identification of dsRNAs based on editing enrichment may yield limited sensitivity. dsRID makes predictions based solely on features in mapped reads, making it possible to examine the potential existence of dsRNAs outside of EERs.

Among the features used in dsRID, skipping ratio and length of the skipped region contributed the most to the model. The skipping ratios of dsRNA regions were generally lower than that of the random control regions. This observation indicates that RT-induced template switching occurs at a low frequency. It should be noted that during RNA isolation and RT, most RNA structures may have denatured and only very strong ones may remain. Thus, the dsRID method is suitable for searches of highly structured regions, such as those formed by EERs. In addition, the strongly structured regions may fold into other types of RNA structures, which may also cause template switching in RT. Nonetheless, the training step of dsRID focuses on dsRNAs formed by EERs, thus enriching for this type of RNA structures. Additionally, dsRID uses RNAfold to check for predicted structures, to further enrich for strong dsRNA structures.

Notably, the skipping ratios of the random controls (defined as random regions with at least six reads and at least one read with region-skipping) showed a bimodal distribution, similar to the distribution of exon inclusion levels of splicing. In addition, compared to EER-based dsRNAs, the random regions were significantly closer to or directly overlapped with spliced junctions. In contrast, the skipping length of EER-based dsRNAs is larger than that of random controls. For random controls, a region of 2500 nt in length was considered, which is shorter than typical introns in human genes. Based on the above observations, the set of random controls may be enriched with both alternatively spliced events with relatively short introns and other skipping events due to sequencing errors/genetic variants or other reasons.

More than 73% of J2 fCLIP-captured regions were also included in dsRID (EER-based on novel predictions), supporting the effectiveness of our approach. Nonetheless, the immunogenic nature of dsRID-predicted dsRNAs need to be experimentally tested in the future. Many dsRNAs identified in our study are located in intronic regions that may not be exported into the cytoplasm, thus may not have any immunogenic effects through cytoplasmic sensors. Previous studies showed that immunogenic dsRNAs are generally depleted and under negative selection in a wide range of species (Barak *et al.* 2020). Thus, we expect cytoplasmic immunogenic dsRNAs may be a small fraction of the dsRID-predicted dsRNAs.

In this study, we focused on developing and applying dsRID using PacBio long-read sequencing data. In general, dsRID can be applied to data generated by other long-read sequencing technologies or short-read RNA-seq data, since the features used in the model can be derived from the other data types as well. However, the impact of different sequencing protocols and RT enzymes on the features and performance of the method should be investigated thoroughly.

Together, we showed that dsRID is an effective method to detect dsRNA regions *in silico*. Our method featured novel dsRNA regions that are lowly edited and may be missed by EER-based approaches. Future studies highlighting long-read sequencing data in different contexts can be analyzed by dsRID to better understand the landscape of dsRNA, its regulation and function.

## Supplementary Material

btad649_Supplementary_Data

## Data Availability

PacBio long-read RNA-seq data of Alzheimer’s disease are available via https://downloads.pacbcloud.com/public/data set/Alzheimer2019_IsoSeq. Long-read RNA-seq data of GM12878 are available via the ENCODE project with accession number ENCSR462COR. Alzheimer’s disease and control brain long-read RNA-seq data are available via the ENCODE project with accession numbers ENCSR169YNI, ENCSR257YUB, ENCSR690QHM, ENCSR316ZTD, ENCSR697ASE, ENCSR094NFM, ENCSR463IDK, and ENCSR205QMF. Genomic coordinates of regions predicted by dsRID in all samples after structural filtering are available at GitHub: https://github.com/gxiaolab/dsRID.

## Code availability

Software implementation of dsRID is available at GitHub: https://github.com/gxiaolab/dsRID. Software implementation to replicate these results has been published in doi: 10.5281/zenodo.8384704.
