# Supplementary material for: dsRID: in silico identification of dsRNA regions using long-read RNA-seq data
Source: Bioinformatics. 2023 Oct 23;39(11):btad649. doi: 10.1093/bioinformatics/btad649 (PMC10628436; doi:10.1093/bioinformatics/btad649)
Supplement: btad649_Supplementary_Data [file btad649_supplementary_data.pdf]

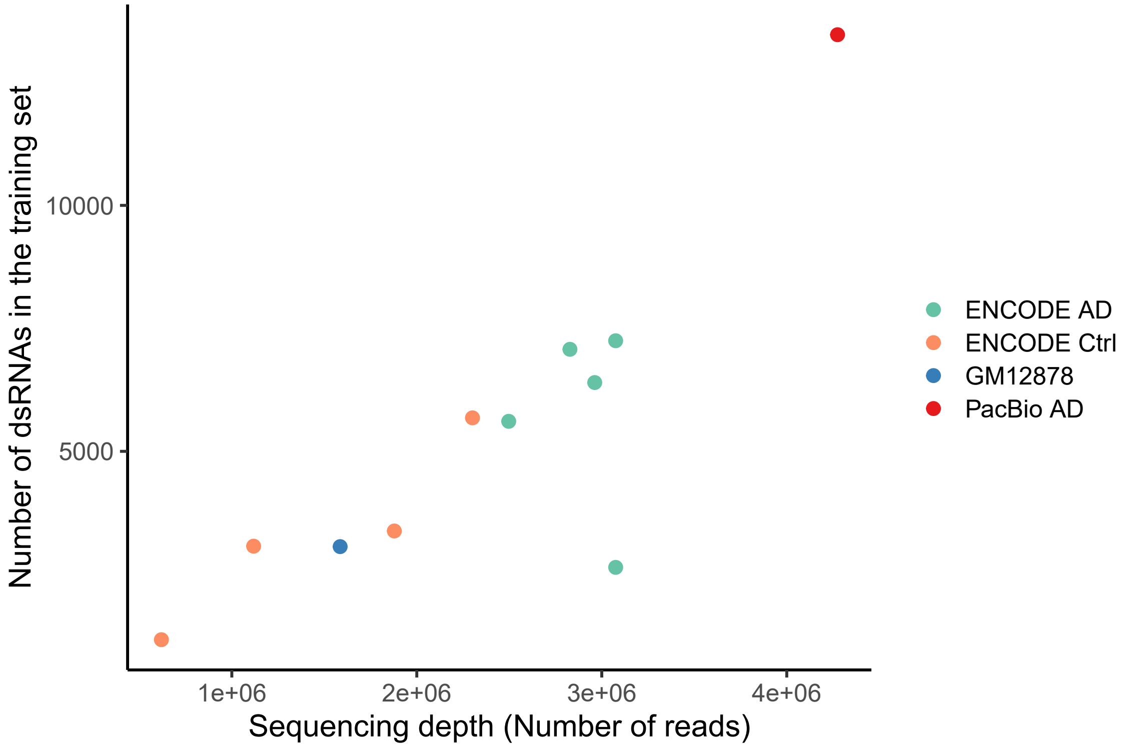

Figure S1: Dot plot showing relationship between sequencing depth and number of dsRNA regions included in training. Color represents cohort type.

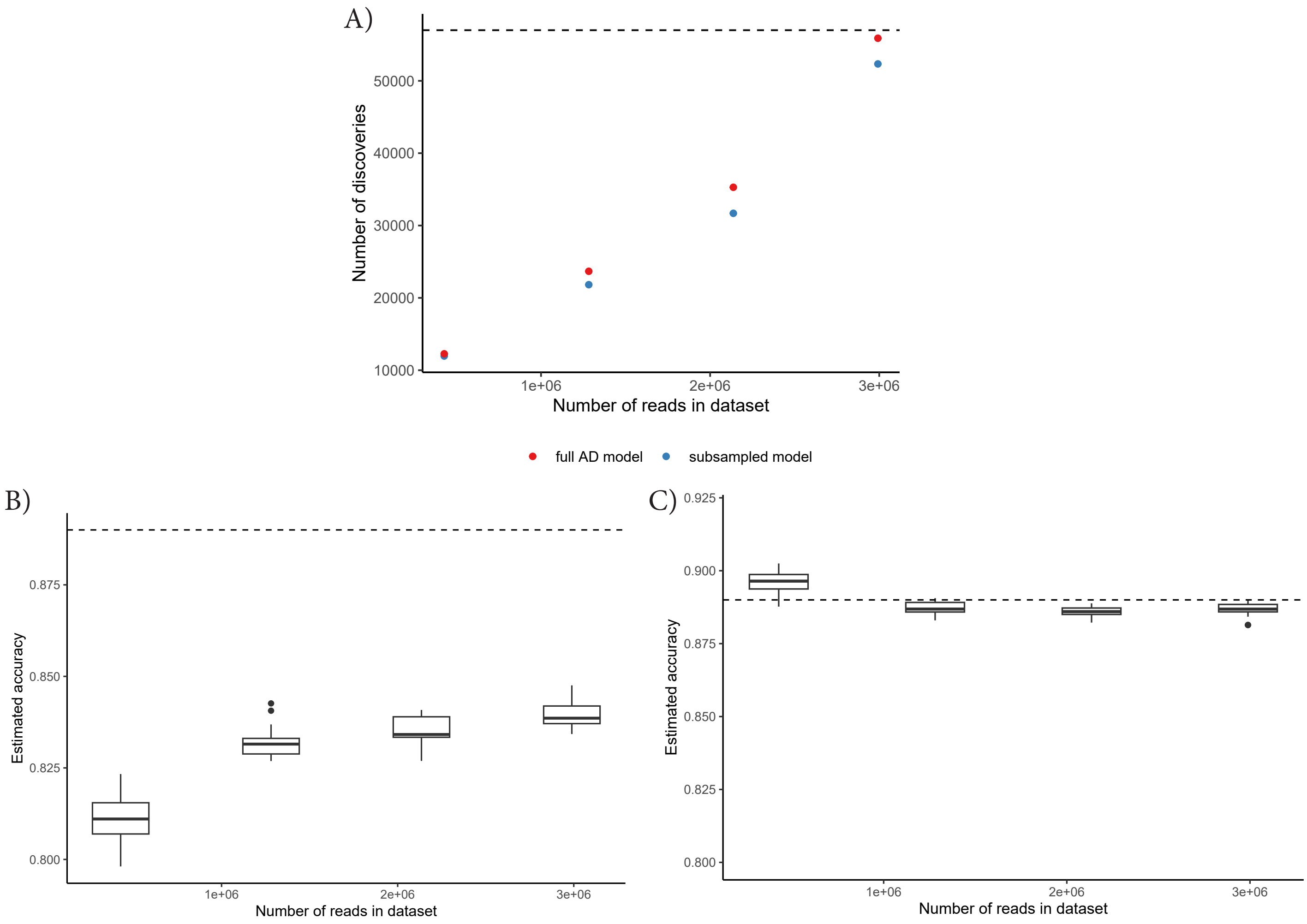

Figure S2: A) Dot plot showing relationship between the number of reads in the dataset and the number of candidate regions discovered when the model was applied to subsampled data sets. The color indicates whether the model was trained on the subsampled dataset or the full PacBio AD data. Dashed line represents the number of discoveries when the model was applied to the full AD dataset. B) Box plot showing estimated accuracy for each subsampled data set. The accuracy was estimated using a random 5% of full PacBioAD data, repeated 20 times, using the model trained with each subsampled data set. Dashed line represents the mean accuracy using the model trained by the full PacBio AD dataset. C) Box plot showing estimated accuracy using the model trained by the full PacBio AD data but applied to a random 5% of each subsampled data set, repeated 20 times.

A)

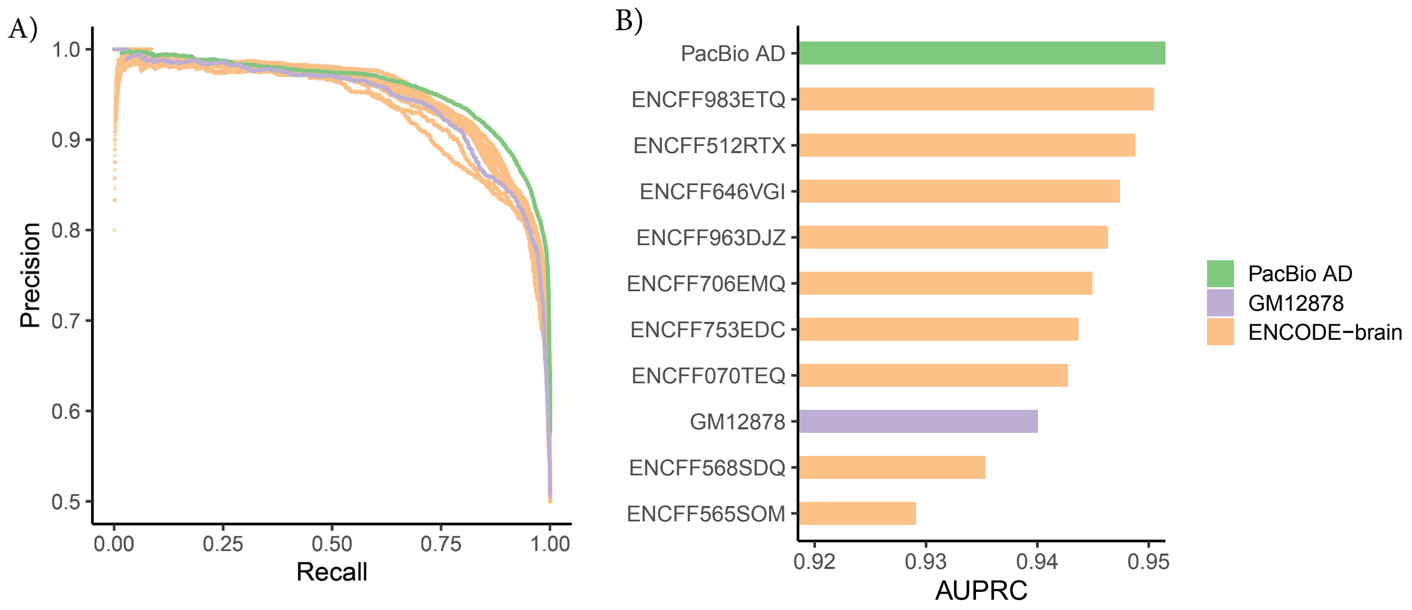

Figure S3: A) Precision-recall curve across different datasets. Color represents dataset type. B) Bar plot showing area under the precision-recall curve scores across different datasets.

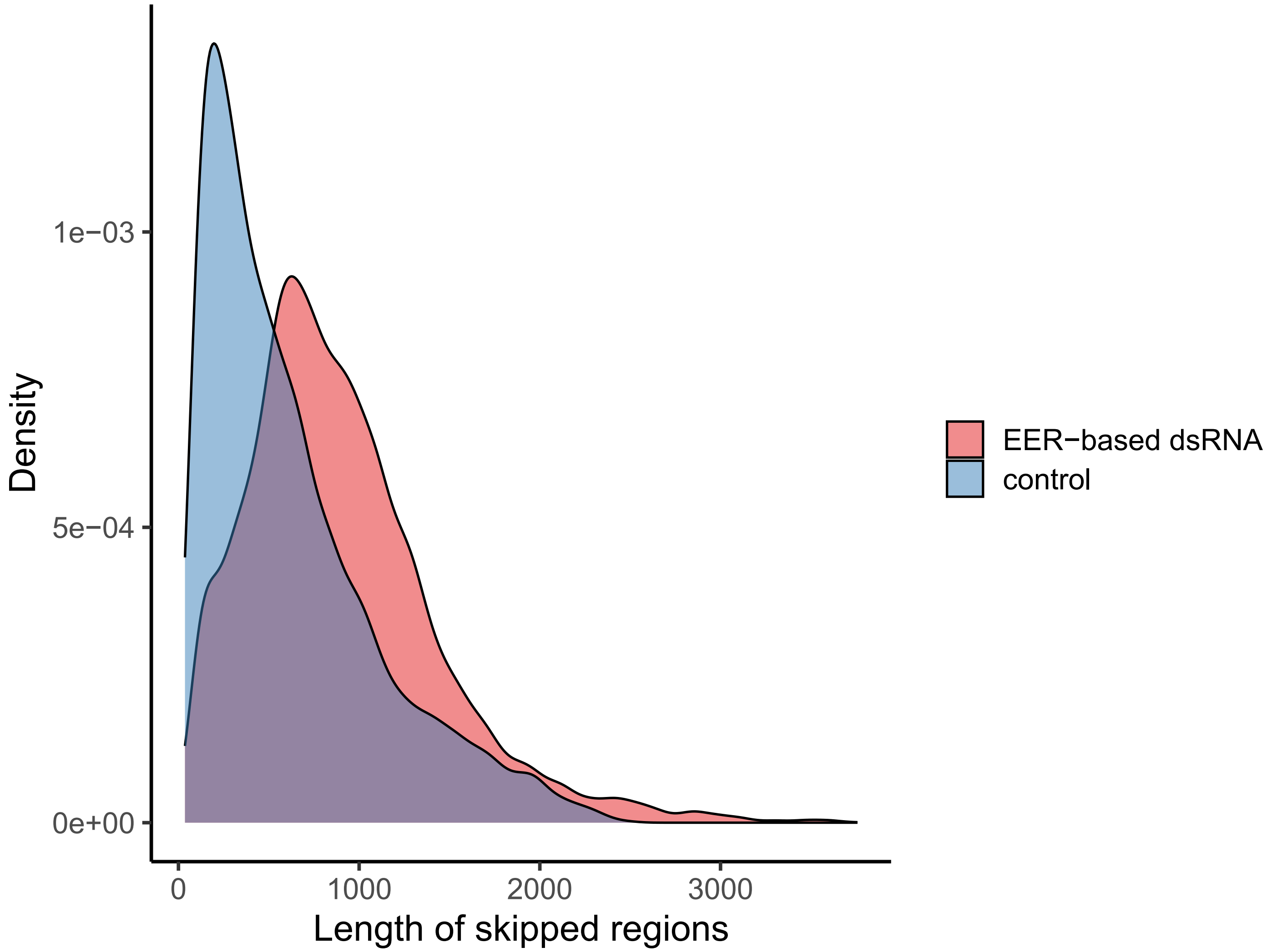

Figure S4: Distribution of skipping length in EER-based dsRNAs and controls.

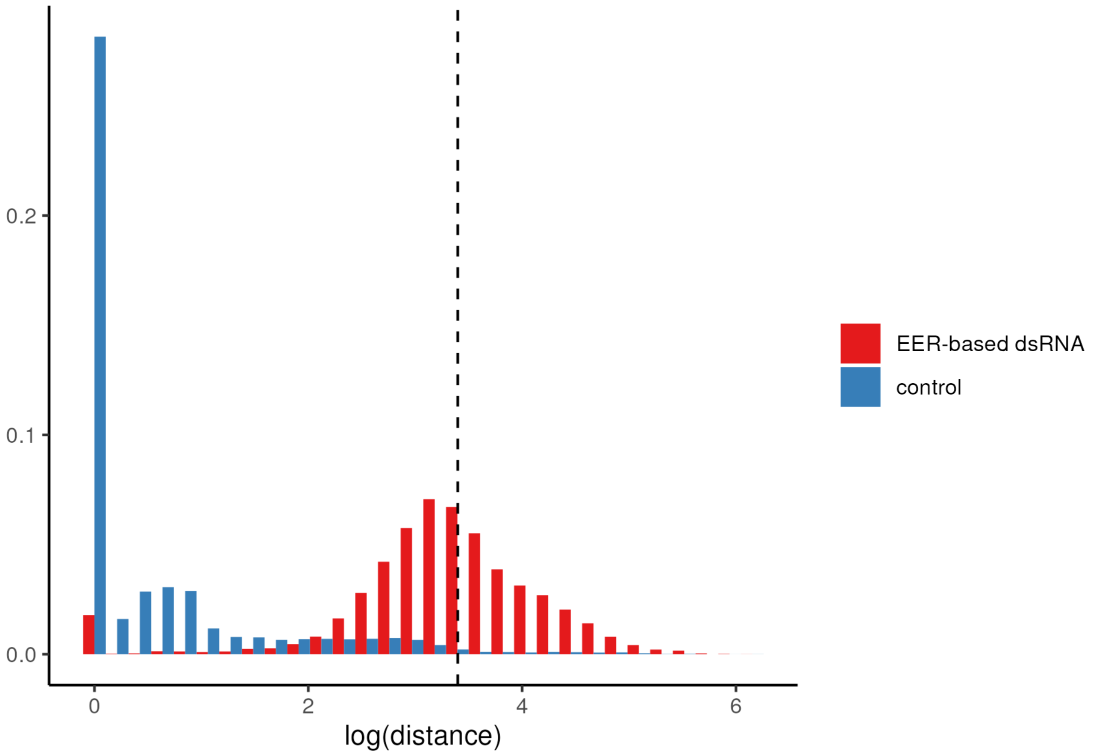

Figure S5: Histogram showing the distribution of distances between skipping sites and closest known splicing junctions in log scale. Colors indicate EER-based dsRNA regions and control regions respectively.

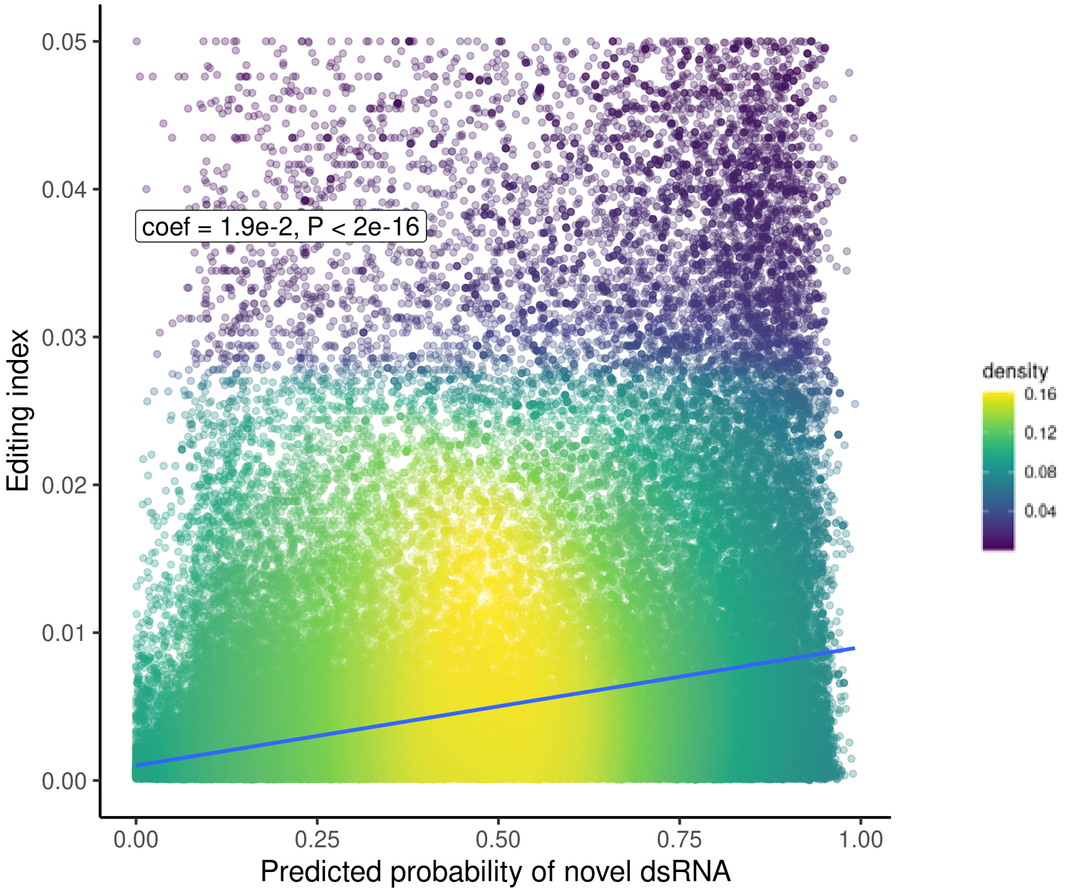

Figure S6: Dot plot showing relationship between editing index and predicted probability. Coefficients and p-values are tested by linear regression model.

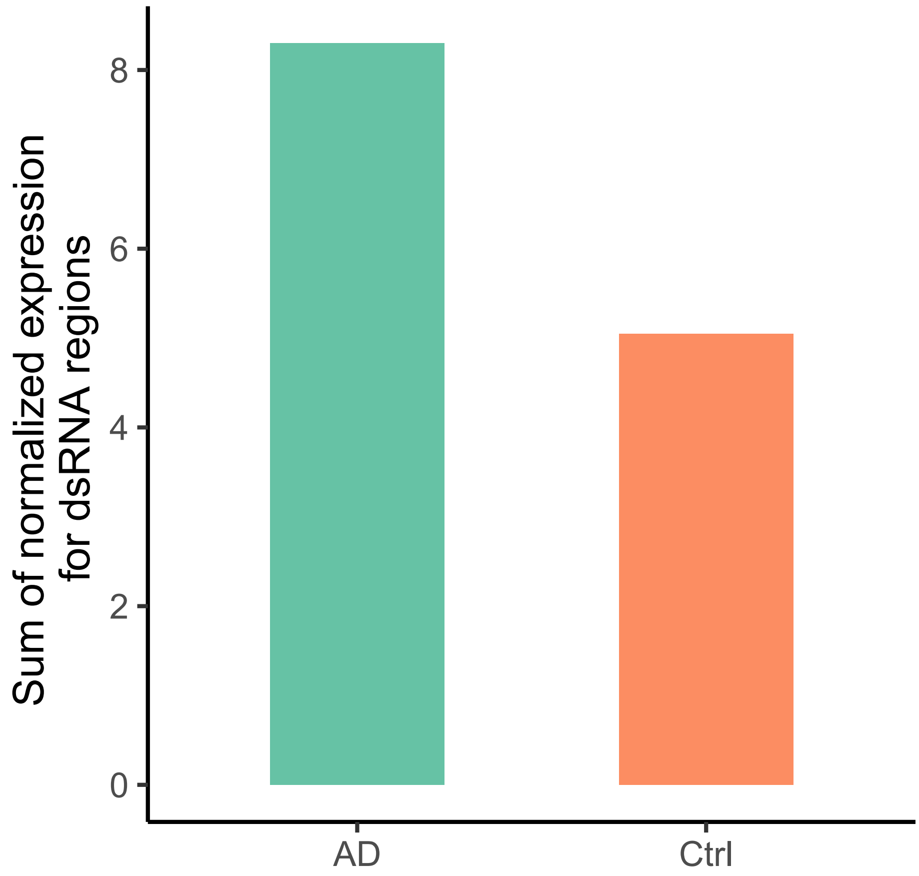

Figure S7: Bar plot showing sum of total expression between disease status. Y-axis represents sum of normalized expression for predicted dsRNAs and x-axis showing disease status.
